# A population-based case-control study of Selective Serotonin Reuptake Inhibitors (SSRIs) and breast cancer: The impact of duration of use, cumulative dose and latency

**DOI:** 10.1186/1741-7015-8-90

**Published:** 2010-12-22

**Authors:** JE Ashbury, LE Lévesque, PA Beck, KJ Aronson

**Affiliations:** 1 Division of Cancer Care and Epidemiology, Cancer Research Institute, 10 Stuart Street, 2nd Level, Queen's University, Kingston, ON K7L 3N6, Canada; 2 Department of Community Health and Epidemiology, Carruthers Hall, Queen's University, Kingston, ON K7L 3N6, Canada; 3 Kingston, Frontenac, Lennox and Addington (KFL&A) Public Health, 221 Portsmouth Avenue, Kingston, ON K7 M 1V5, Canada; 4 Population Health Branch, Saskatchewan Ministry of Health, T.C. Douglas Building, 3475 Albert Street, Regina, SK S4 S 6X6, Canada

## Abstract

**Background:**

Selective serotonin reuptake inhibitors (SSRIs), a popular class of antidepressants, may increase breast cancer risk by stimulating the secretion of prolactin, a potential tumour promoter. We evaluated the effects of duration of SSRI use, cumulative dose, and latency on the risk of breast cancer by conducting a population-based case-control study utilizing Saskatchewan health databases.

**Methods:**

Cases included 1,701 women with primary invasive breast cancer diagnosed from 2003 to 2006, and controls consisted of 17,017 women, randomly selected from the population registry. Use of SSRIs was compiled using the Saskatchewan prescription database. Unconditional logistic regression was conducted to evaluate the impact of duration of combined SSRI use (total number of prescriptions dispensed), cumulative dose (total dosage received) and timing of use (two or more years, two to seven years and more than seven years prior to index date) on the risk of breast cancer.

**Results:**

Overall, SSRI use was not associated with an increased risk of breast cancer regardless of our definition of cumulative use (total number of prescriptions dispensed and total dosage). In addition, our results indicate that prolonged SSRI use does not have a latent effect on breast cancer risk. Also, our findings are not suggestive of an increased risk of breast cancer with the use of individual SSRIs.

**Conclusions:**

Our study improved upon most previous studies by having a longer follow-up period, a larger sample size of long-term SSRI users and consideration of risk during specific exposure time windows that take latency into account. Given the potential health benefits of using SSRIs, our results suggest that the issue of breast cancer risk may no longer be a concern for women requiring long-term SSRIs.

## Background

Experimental evidence suggests that SSRIs, a popular class of antidepressants, enhance breast cell proliferation during a relatively late phase in breast cancer development, either directly through SSRI tumour-promoting mechanisms [1-3] or indirectly through an SSRI-mediated increase in prolactin, a hormone for which there is increasing evidence to suggest an association with breast cancer [4,5].

Previous epidemiologic studies examining the relationship between SSRI use and breast cancer were mainly conducted in the 1990's when the prevalence of SSRI use was low and therefore those studies were limited by a lack of subjects with prolonged use of SSRIs [6]. As such, few epidemiologic studies have investigated the long-term safety of SSRIs or considered a latent effect of SSRI use on breast cancer risk. In a review, Coogan [6] highlighted the 'dearth of data' related to long-term SSRI use and breast cancer risk and recommended further study. Given that breast cancer is the most frequently diagnosed cancer in women [7,8] and that SSRIs have now been on the market for more than 20 years and are increasingly widely prescribed particularly in women, further investigation of the potential association between long-term SSRI use and breast cancer risk is warranted.

In this study, we evaluated the effects of duration of use, cumulative dose, and latency on breast cancer risk using a population-based case-control study.

## Methods

### Data sources and linkage

This population-based case-control study utilized three administrative health databases from the province of Saskatchewan (SK), Canada: (1) the person registry system (PRS) (population registry) for information on demographics and dates of health coverage for the majority of SK residents, (2) the Saskatchewan prescription drug plan (SPDP) database for all outpatient prescriptions covered by the drug plan filled since the mid-1970's for most SK residents, and (3) the Saskatchewan Cancer Agency (SCA) cancer registry database for detailed information on cancer diagnoses since 1970 [9]. Information from all three databases was available for approximately 91% of residents (about one million people) since the remainder have their prescription drug benefits covered by federal government agencies. In Saskatchewan, the Health Services Number (HSN) is a lifetime number that uniquely identifies each resident eligible for provincial health insurance coverage and enables complete record linkage across databases and over time [9].

### Selection of cases and controls

The underlying study population for cases and controls was conceptualized as all women eligible for outpatient prescription drug benefits who, during the case accrual period 2003 to 2006, were aged 28 to 79, had prescription coverage for 10 or more consecutive years prior to their index date, and no previous cancer diagnosis at any site in the 10 years preceding the index date, with the exception of non-melanoma skin cancer and *in situ *cervical cancer. We included all incident cases of primary invasive breast cancer, diagnosed between 1 January 2003 and 31 December 2006, who met the eligibility criteria listed above. Controls were selected from the underlying study population using an incidence density (or risk-set) sampling approach [10]. Briefly, the four-year case accrual period was divided into eight sampling periods each of six months duration to define the risk sets from which women who were still at risk of the event (controls) were chosen. Ten controls per case, frequency-matched on cases' age in five-year groups, were randomly selected from each risk set. The index date for cases was the date of breast cancer diagnosis, and for controls it was a randomly chosen date within each six-month sampling period.

We initially identified 1,702 women diagnosed with breast cancer (1,699 (99.8%) primary invasive cancers and 3 (0.2%) carcinomas *in situ*), (the latter were retained in the analysis because of their small numbers) between 1 January 2003 and 31 December 2006 and 17,020 matched controls. One case and three controls were excluded from the analysis because of missing demographic data leaving 1,701 cases and 17,017 controls.

### SSRI use

Information was obtained on all SSRI prescriptions dispensed for cases and controls between the start of their drug plan coverage or 1 July 1989 (when the first SSRI was listed on the SK Formulary), whichever was later, and the index date. SSRIs covered by the provincial drug plan during the study period included citalopram, fluoxetine, fluvoxamine, paroxetine and sertraline and were available with no prescribing restrictions. SSRIs were first considered as a group, then as individual agents. Data extracted from the prescription drug database included the dispensing date, name, strength, dosage formulation, and quantity of the SSRI dispensed. The prescription database did not include information on the dosage prescribed (pills per dose and total number of doses per day), the duration of each prescription, or the indication for the SSRI prescription.

The observation period was divided into three exposure time windows of interest. First, breast cancer risk was examined in relation to total SSRI use during the exposure time period two or more years prior to index date thereby representing a two-year latency between SSRI use and breast cancer diagnosis. SSRI use within two years of index date was not considered etiologically relevant and was therefore excluded from the analysis similar to previous studies [11,12], and supported by models of tumour growth [13] and experimental laboratory evidence of an SSRI promoter effect on breast cancer development [1]. In addition, exclusion of this biologically irrelevant SSRI exposure period minimized the potential for reverse causality bias (when SSRI use is associated with cancer that is already present but not clinically detectable).

To further evaluate the effect of latency on the association between SSRI use and breast cancer risk, the exposure time window two or more years prior to the index date was subdivided into two shorter mutually exclusive exposure time windows chosen *a priori *(two to seven years preceding index date representing a two-year latency and more than seven years prior to index date representing a latency of seven years). We hypothesized that either prolonged SSRI use within the time period two to seven years or more than seven years prior to index date may be associated with increased risk, associations that may be missed if risk was examined only in relation to SSRI use over the exposure time window two or more years prior to index date. The comparator group for each of these analyses was women with no SSRI prescriptions (non-use of SSRIs) during the specific exposure time window being analyzed.

Two measures of cumulative SSRI use were used: duration (total number of prescriptions dispensed) and cumulative dose (total dosage received). These were modelled as categorical variables in order to facilitate the analysis of the most clinically relevant contrast, that is, long-term users versus non-users.

Duration of use for all SSRIs combined was based on the total number of SSRI prescriptions dispensed in each of the SSRI exposure time windows of interest (two or more years, two to seven years or more than seven years prior to index date). Since SSRI prescriptions in Saskatchewan are typically dispensed to accommodate a 34-day treatment period, this measure of SSRI use approximated the number of months exposed. For each exposure time period, duration of SSRI use was categorized on the basis of clinically relevant cut-offs with the intention of creating an uppermost category of SSRI users that included an adequate number of subjects. For the time periods two or more years and two to seven years prior to the index date, duration of SSRI was categorized as never, 1 to 35 prescriptions and ≥36 prescriptions. Due to the smaller number of subjects who had used SSRIs in the period of time more than seven years prior to index date, exposure categories chosen were never, 1 to 23 and ≥24 prescriptions.

The cumulative dose of SSRIs was calculated in terms of fluoxetine-equivalent milligrams for each prescription using the defined daily doses (DDD) published by the World Health Organization [14] and summed across all prescriptions. The DDD is a unit of measurement that allows comparisons of therapeutic intensity between medications from the same therapeutic class [15]. The DDD fluoxetine-equivalent dosages for the five SSRIs included in this study were: 20 milligrams (mg) of fluoxetine = 20 mg of citalopram = 20 mg of paroxetine = 50 mg of sertraline = 100 mg of fluvoxamine [14]. Although it may be debated whether fluoxetine-equivalent dosages are representative of the carcinogenic potential of individual agents, this measure of SSRI use was chosen so that the cumulative dose could be interpreted in the context of clinically relevant dosages. Total dosages were categorized as no use, low dose (< 85^th ^percentile) and high dose (≥85^th ^percentile) based on the distribution of total dosages among exposed controls for each of the three exposure time windows. This cut-off was chosen to identify a comparable group of highly exposed individuals to that used in the duration of use (number of prescriptions) analysis.

For the analysis of individual SSRIs, duration of use was represented by the total number of prescriptions dispensed and limited to SSRI use two or more years prior to index date because of small sample sizes. 'Any use' referred to women who had used the individual SSRI of interest alone or in combination with one or more other SSRIs, whereas 'exclusive use' referred to the subset of 'any use' who had used only one type of SSRI during the study period.

### Potential confounders

Using yearly demographic data available from the population registry, subjects were categorized for marital status (married, single, other), residence status (urban, rural) and income support status (no support, low support, and high support) (See Table 1 footnotes for details). As previously mentioned, we frequency-matched on age at index date in five-year groups and adjusted for age (continuous).

Medication history for use of oral contraceptives (OCs) and hormone therapy (HT) was compiled for the period between (a) 1 September 1975 (the year that the SPDP database was started) or (b) the date of prescription coverage initiation, (whichever occurred later), and the subject's index date (See Table 1 footnotes for details). OC and HT prescription data were not available from July 1987 to December 1988 when pharmacy data were recorded by family unit rather than by the individual [9]. Consistent with the approach taken for SSRI exposures, use of OCs and HT within two years of the index date was excluded.

### Statistical analysis

Unconditional logistic regression was conducted to evaluate the impact of duration of combined SSRI use (total number of prescriptions dispensed), cumulative dose (total dosage received) and timing of use (two or more years, two to seven years and more than seven years prior to index date) on the risk of breast cancer. For each individual SSRI, duration of use (total number of prescriptions dispensed) was analyzed only for the time period two or more years prior to index date. To evaluate potential confounders, the 'change in estimate' method was used with a ≥10% criterion for inclusion [16,17]. No covariate tested (age - continuous, marital status, residence, income support status, oral contraceptive and hormone therapy use - categorical) met the definition of a confounder. However, we chose to use a fully adjusted model for each analysis since there was no loss of precision compared to the parsimonious model.

## Results

The study population consisted of 1,701 cases and 17,017 controls. Cases were diagnosed at a mean age of 60.4 years (SD 11.5) and represented a homogeneous group with respect to morphology, with approximately 75% having infiltrating ductal carcinoma, 10% lobular carcinoma, and 15% with a mix of other morphologies. There was more variability in relation to stage at diagnosis, with 44.9% of cases presenting as stage one, 35.3% stage two, 12.6% stage three, 4.5% stage four, 0.2% *in situ *and 2.5% unknown (stage could not be assessed). The proportion of breast cancer cases diagnosed each year was similar across the four-year accrual period.

Cases and controls were followed for an average of 28.4 years and were similar with regard to age, income support, and residence status (Table 1). However, cases were more likely than controls to be married and to have used hormone therapy and/or oral contraceptives.

**Table 1:** Characteristics of cases and age-matched controls

Characteristic	Cases (N = 1701)		Controls (N = 17017)		**Unadjusted OR **^**a**^ (95% CI)
	n	(%)	n	(%)	
**Age (yrs)**					
Mean (SD)	60.4	(11.5)	60.3	(11.6)	1.00 (1.00, 1.01)
					
**Age categories**					
<40 (%)	50	(2.9)	500	(2.9)	1.00
40-55 (%)	540	(31.8)	5500	(32.3)	1.01 (0.75, 1.36)
>55 (%)	1111	(65.3)	11017	(64.7)	0.98 (0.73, 1.33)
					
**Follow-up (yrs) ^b^**					
Mean (SD)	28.4	(3.3)	28.4	(3.5)	1.03 (1.00, 1.06)
Median (range)	29	(10,31)	29	(10,31)	
					
**Marital Status ^c^**					
Other (widowed, separated, divorced)	309	(18.2)	3565	(21.0)	1.00
Single	108	(6.4)	1111	(6.5)	1.12 (0.89, 1.41)
Married	1284	(75.4)	12341	(72.5)	1.20 (1.05, 1.37)
					
**Income support status ^d^**					
No income support	1232	(72.4)	12088	(71.0)	1.00
Low income support	412	(24.2)	4235	(24.9)	0.96 (0.85, 1.07)
High income support	57	(3.4)	694	(4.1)	0.81 (0.61, 1.06)
					
**Residence status ^e^**					
Urban	731	(43.0)	6984	(41.0)	1.00
Rural	970	(57.0)	10033	(59.0)	0.92 (0.84, 1.02)
					
**OC use ^f^**					
None or light use (<12 prescriptions)	1318	(77.5)	13557	(79.7)	1.00
Heavy use (≥ 12)	383	(22.5)	3460	(20.3)	1.14 (1.01, 1.28)
					
**HT use ^f^**					
None or light use (<12 prescriptions)	1200	(70.6)	12441	(73.1)	1.00
Heavy use (≥12)	501	(29.4)	4576	(26.9)	1.14 (1.02, 1.27)

OR = odds ratio; CI = confidence interval; OCs = oral contraceptive(s); HT = hormone therapy.

^a ^Frequency-matched on age in five-year groups but not adjusted for the influence of other risk factors.

^b ^Follow-up: Number of years eligible to receive outpatient prescription drug benefits in Saskatchewan.

^c ^Marital status: attributed to the marital state six years prior to index date.

^d ^Income support status: based on yearly demographic data and assigned to one of three categories based on the greatest proportion of time having received income support: that is, no income support, ≤50% of time receiving income support = low income support, and >50% of time receiving income support = high income support.

^e ^Residence status: based on yearly demographic data and assigned based on the greatest proportion of time spent in a given residence state: that is, ≤50% of time in urban location (population >100,000) = rural residence status and >50% of time as urban = urban residence status.

^f ^OC and HT use: defined as total number of prescriptions two or more years prior to index date. These drugs are typically dispensed as a two-month supply; therefore, in most cases, 12 prescriptions would correspond to a two-year supply. All oral and injectable contraceptives were included as were all hormone therapy formulations except vaginal creams and rings.

**Note: **Percentages may not add up to 100% due to rounding.

Table 2 shows the unadjusted and adjusted ORs and 95% CIs for breast cancer risk according to the number of SSRI prescriptions dispensed for all SSRIs combined during each of three exposure time windows of interest (two or more years, two to seven years and more than seven years prior to index date). After adjustment for risk factors, women who had filled ≥36 prescriptions (estimated SSRI use of three or more years) in the time period two or more years prior to index date were not at increased risk of developing breast cancer compared to non-users (OR = 1.03, CI = 0.75 - 1.41). Similarly, long-term use of SSRIs during the exposure time period two to seven years or more than seven years before the index date was not associated with an increased risk of breast cancer compared to non-users (OR = 1.13, CI = 0.78 - 1.64, and OR = 0.80, CI = 0.49 - 1.29, respectively). A shorter duration of use was also not associated with an increased risk of breast cancer regardless of the time period evaluated.

**Table 2:** Odds ratios for breast cancer according to duration of SSRI use during exposure time windows

**Exposure time window **^**a**^	No. of SSRI prescriptions	Cases (n = 1,701)		Controls (n = 17,017)		**Unadjusted OR **^**b**^ (95% CI)	**Adjusted OR **^**c**^ (95% CI)
		n	(%)	n	(%)		
≥2 years	No use ^d ^(reference)	1,442	(84.8)	14,415	(84.7)	1.00	1.00
	1 to 35	215	(12.6)	2,178	(12.8)	0.99 (0.85, 1.15)	0.98 (0.84, 1.14)
	≥36	44	(2.6)	424	(2.5)	1.04 (0.76, 1.42)	1.03 (0.75, 1.41)
							
2 to 7 years	No use ^d ^(reference)	1,512	(88.9)	15,122	(88.9)	1.00	1.00
	1 to 35	157	(9.2)	1,614	(9.5)	0.97 (0.82, 1.16)	0.97 (0.82, 1.15)
	≥36	32	(1.9)	281	(1.7)	1.14 (0.79, 1.65)	1.13 (0.78, 1.64)
							
>7 years	No use ^d ^(reference)	1,556	(91.5)	15,528	(91.3)	1.00	1.00
	1 to 23	127	(7.5)	1,266	(7.4)	1.00 (0.83, 1.21)	1.00 (0.82, 1.21)
	≥24	18	(1.0)	223	(1.3)	0.81 (0.50, 1.31)	0.80 (0.49, 1.29)

OR = odds ratio; CI = confidence interval

^a ^Prior to index date.

^b ^Matched on age in five-year age groups but unadjusted for the influence of other risk factors.

^c ^Adjusted for age at index (years), marital status, income support status, residence status, and use of oral contraceptives and/or use of hormone therapy.

^d ^'No use' (reference category) refers to women with no SSRI prescriptions dispensed during the specified time period evaluated.

**Note: **Percentages may not add up to 100% due to rounding.

Compared with no use of SSRIs, higher total dosages (based on fluoxetine-equivalent dosages for all SSRIs combined) did not increase the risk of breast cancer during each exposure time window of interest, that is, two or more years prior to index date (OR = 0.91, CI = 0.64 - 1.28), two to seven years prior to index date (OR = 0.92, CI = 0.62 - 1.36) and use more than seven years prior to index date (OR = 1.02, CI = 0.67 - 1.55) (Table 3). Similarly, lower dosages were not associated with an increased risk in any time period assessed.

**Table 3:** Odds ratios for breast cancer according to cumulative SSRI dose during exposure time windows

**Exposure time window **^**a**^	**Total SSRI dosage **^**b **^ (mg)	Cases (n = 1,701)		Controls (n = 17,017)		**Unadjusted OR **^**c**^ (95% CI)	**Adjusted OR **^**d**^ (95% CI)
		n	(%)	n	(%)		
≥2 years	No use ^e ^(reference)	1,442	(84.8)	14,415	(84.7)	1.00	1.00
	1 to 27, 750 mg	222	(13.1)	2,198	(12.9)	1.01 (0.87, 1.17)	1.01 (0.87, 1.17)
	≥27, 750 mg	37	(2.2)	404	(2.4)	0.92 (0.65, 1.29)	0.91 (0.64, 1.28)
							
2 to 7 years	No use ^e ^(reference)	1,512	(88.9)	15,122	(88.9)	1.00	1.00
	1 to 25, 365 mg	161	(9.5)	1,594	(9.4)	1.01 (0.85, 1.20)	1.01 (0.85, 1.20)
	≥25, 365 mg	28	(1.7)	301	(1.8)	0.93 (0.63, 1.38)	0.92 (0.62, 1.36)
							
>7 years	No use ^e ^(reference)	1,558	(91.6)	15,475	(90.9)	1.00	1.00
	1 to 20, 340 mg	118	(6.9)	1,303	(7.7)	0.90 (0.74, 1.09)	0.90 (0.74, 1.09)
	≥20, 340 mg	25	(1.5)	239	(1.4)	1.04 (0.69, 1.57)	1.02 (0.67, 1.55)

OR = odds ratio; CI = confidence interval

^a ^Prior to index date.

^b ^Combined dosage calculated using fluoxetine-equivalent dosages according to the World Health Organization's defined daily doses (DDD) methodology.

^c ^Matched on age in five-year age groups but unadjusted for the influence of other risk factors.

^d ^Adjusted for age at index (years), marital status, income support status, residence status, and use of oral contraceptives and/or use of hormone therapy.

^e ^'No use' (reference category) refers to women with no SSRI prescriptions dispensed during the specified time period evaluated.

**Note: **Percentages may not add up to 100% due to rounding.

The pattern of risk observed with the use of individual SSRIs was similar to that for all SSRIs combined (Table 4 and 5). Overall, there was no evidence of increased risk of breast cancer associated with 'any use' of individual SSRIs, regardless of the total number of prescriptions dispensed (Table 4). The small number of users of specific SSRIs did not permit an evaluation of risk associated with their use during the two shorter exposure windows. The odds ratios for 'exclusive use' of high doses of paroxetine (OR = 1.20, CI = 0.64 - 2.25), sertraline (OR = 1.84, CI = 0.86 - 3.93) and fluvoxamine (OR = 1.33, CI = 0.47 - 3.77) failed to rule out the possibility of an increased risk; however, the number of exclusive users of any one agent was small and the corresponding confidence intervals very wide (Table 5).

**Table 4:** Odds ratios for breast cancer according to duration for 'any use' of individual SSRIs

No. of SSRI prescriptions	**Any use **^**a**^					
	
	Cases		Controls		**Unadjusted OR **^**b**^ (95% CI)	**Adjusted OR **^**c**^ (95% CI)
	n	(%)	n	(%)		
**No use ^d ^**(reference)	1,442	(84.8)	14,415	(84.7)	1.00	1.00

**Paroxetine**						
1 to 23	94	(6.0)	893	(5.8)	1.05 (0.85, 1.31)	1.05 (0.84, 1.31)
≥24	20	(1.3)	184	(1.2)	1.09 (0.68, 1.73)	1.06 (0.67, 1.69)

**Sertraline**						
1 to 23	46	(3.1)	518	(3.5)	0.89 (0.65, 1.21)	0.89 (0.65, 1.21)
≥24	10	(0.7)	103	(0.7)	0.97 (0.51, 1.86)	0.98 (0.51, 1.89)

**Fluoxetine**						
1 to 23	82	(5.3)	893	(5.8)	0.92 (0.72, 1.64)	0.92 (0.71, 1.64)
≥ 24	25	(1.6)	231	(1.5)	1.08 (0.71, 1.64)	1.08 (0.71, 1.64)

**Fluvoxamine**						
1-23	45	(3.0)	411	(2.8)	1.10 (0.80, 1.50)	1.10 (0.80, 1.51)
≥24	4	(0.3)	52	(0.4)	0.77 (0.28, 2.13)	0.78 (0.28, 2.18)

**Citalopram**						
1 to 23	27	(1.8)	314	(2.1)	0.86 (0.58, 1.28)	0.85 (0.57, 1.27)
≥24	3	(0.2)	23	(0.2)	1.30 (0.39, 4.35)	1.28 (0.38, 4.27)

OR = odds ratio; CI = confidence interval

^a ^'Any use' refers to use of individual SSRI of interest alone or in combination with one or more other SSRIs during the exposure time window two or more years prior to index date.

^b ^Matched on age in five-year age groups but unadjusted for the influence of other risk factors.

^c ^Adjusted for age at index (years), marital status, income support status, residence status, and use of oral contraceptives and/or use of hormone therapy.

^d ^'No use' (reference category) refers to women with no SSRI prescriptions dispensed two or more years prior to index date.

**Table 5:** Odds ratios for breast cancer according to duration for 'exclusive use' of individual SSRIs

No. of SSRI prescriptions	**Exclusive use **^**a**^					
	
	Cases		Controls		**Unadjusted OR **^**b**^ (95% CI)	**Adjusted OR **^**c**^ (95% CI)
	n	(%)	n	(%)		
**No use ^d ^**(reference)	1,442	(84.8)	14,415	(84.7)	1.00	1.00

**Paroxetine**						
1 to 23	57	(3.8)	482	(3.2)	1.18 (0.89, 1.56)	1.17 (0.88, 1.55)
≥24	11	(0.73)	89	(0.6)	1.24 (0.66, 2.32)	1.20 (0.64, 2.25)

**Sertraline**						
1 to 23	22	(1.5)	240	(1.6)	0.92 (0.59, 1.42)	0.92 (0.59, 1.42)
≥24	8	(0.5)	44	(0.3)	1.82 (0.85, 3.87)	1.84 (0.86, 3.93)

**Fluoxetine**						
1 to 23	38	(2.6)	483	(3.2)	0.79 (0.56, 1.10)	0.79 (0.56, 1.10)
≥24	1	(0.7)	134	(0.9)	0.82 (0.44, 1.52)	0.81 (0.44, 1.51)

**Fluvoxamine**						
1 to 23	18	(1.2)	174	(1.2)	1.03 (0.64, 1.69)	1.02 (0.63, 1.67)
≥24	4	(0.3)	30	(0.2)	1.33 (0.47, 3.79)	1.33 (0.47, 3.77)

**Citalopram ^e^**						
1 to 23	9	(0.6)	133	(0.9)	Not estimatable	Not estimatable
≥24	0	(0.0)	9	(0.1)		

OR = odds ratio; CI = confidence interval

^a ^'Exclusive use' refers to use of only one type of SSRI during the exposure time window two or more years prior to index date.

^b ^Matched on age in five-year age groups but unadjusted for the influence of other risk factors.

^c ^Adjusted for age at index (years), marital status, income support status, residence status, and use of oral contraceptives and/or use of hormone therapy.

^d ^'No use' (reference category) refers to women with no SSRI prescriptions dispensed two or more years prior to index date.

^e ^Small sample sizes did not allow a complete evaluation of risk for 'exclusive use' of citalopram.

## Discussion

This large population-based case-control study utilized administrative health databases to assess SSRI use and breast cancer risk in relation to duration of use, cumulative dose and latency; critical considerations within the context of a carcinogenic hypothesis [18]. Overall, SSRI use was not associated with an increased risk of breast cancer regardless of our definition of cumulative use (total number of prescriptions dispensed and total dosage). In addition, our results indicate that prolonged SSRI use does not have a latent effect on breast cancer risk. Also, our findings are not suggestive of an increased risk of breast cancer with the use of individual SSRIs.

Our study improved upon most previous studies by having a longer follow-up period (subjects were followed for up to 18 years after initial SSRI exposure) and prospectively collected drug information that afforded precise and objective exposure definitions. Further, few previous studies assessed risk associated with long-term use of SSRIs of comparable duration (that is, the equivalent of three or more years of use) or during specific exposure time windows that take latency into account. However, despite these methodological improvements, our results are consistent with most other observational studies, including six that used self-reported SSRI exposure information [19-24], and four studies that ascertained SSRI use from a prescription database [25-28]. However, only three of these studies [22,24,27] had sufficient sample sizes to adequately evaluate risk associated with three or more years of combined SSRI use, and only one study [27] quantified total SSRI use with a dosage variable.

Haukka *et al*. [29] in a record-linkage study investigating cancer incidence at 19 sites in relation to the use of several antidepressants, reported an RR of 1.53 (CI = 1.14 - 2.05) for breast cancer for subjects with more than four years of SSRI use compared to non-users, adjusted for age and sex. However, this result may be due to residual confounding or Type 1 error since more than 275 analyses were performed.

No previous peer-reviewed published studies have considered a delayed effect of SSRI exposure using similar time periods. However, results from other studies that assessed the effect of timing of SSRI use on breast cancer risk using less precise measures of past SSRI use, such as 'time since first use' or 'time since last use' [19,20,28] or 'current' versus 'former' users [22-24,27] are consistent with our null findings.

Our findings of no increased risk of breast cancer for long-term use of individual SSRIs were consistent with most other observational studies that specifically assessed these relationships [22,23,26-28], but contrary to a non-significant dose-response decrease in risk associated with increasing duration of paroxetine use reported by Wernli *et al*. [24] (*P*-trend = 0.10). Although the associations for 'exclusive use' of high doses of paroxetine, sertraline and fluvoxamine were inconclusive, it is important to remember that the confidence intervals were wide and less than 1% of women in our study had used these agents exclusively for two years or more, suggesting that these results are likely not a source of concern.

The validity of our study is high for several reasons. The Saskatchewan databases are recognized as valuable resources for drug utilization review and pharmacoepidemiologic studies [9,30] with built-in audit and eligibility checks and good cross database integrity for the conditions studied [9]. The advantages of using a prescription database compared to self-reported SSRI use for drug exposure information include relatively easy access to large sample sizes and elimination of exposure recall error and bias [9,31,32]. In addition, with the use of detailed prescription database exposure histories, more precise SSRI exposure measures were calculated to represent duration and dosage of long-term use, allowing us to assess the latent effects of chronic long-term SSRI use more accurately. Lack of selection bias is also a major strength of this study since there is virtually complete case ascertainment, and controls had a known and equal probability of being randomly selected from the same underlying population as the cases during the same time interval.

By focusing on assessing risk associated mainly with long-term SSRI use, it was anticipated that misclassification due to non-compliance (primarily overestimation of exposure) would more likely occur within the low exposure categories (one or two prescriptions filled), rather than in the contrast of most interest (for example, ≥36 prescriptions versus no exposure). That is, if prescriptions were repeatedly refilled, we assume that they were more likely to be consumed. Therefore, especially within the higher exposure group, misclassification of non-users of SSRIs as users would be less likely. Further, non-compliance with dispensed medications would likely affect cases and controls equally in this study, leading to non-differential misclassification, if any.

The Saskatchewan prescription database lacked information on SSRI medications dispensed in hospitals or as samples from physicians [9]. Also, escitalopram, the newest SSRI to become available in Canada (since late 2005), was not included in the SK Drug Formulary at the time of data collection. However, it was anticipated that the degree of potential misclassification (underestimation of exposure) associated with these additional sources of information bias would be uniform across the case and control groups. Also, since the prescription database does not include information on the prescribed daily dosage regime, the total number of prescriptions dispensed was used to estimate duration of SSRI use based on the assumption that each prescription represented a 34-day supply. To indirectly test the extent of misclassification related to estimating duration of exposure using number of prescriptions, risk was estimated for total dosage of combined SSRI use: none was detected, confirming that exposure classification errors in relation to using number of prescriptions were unlikely.

The SK databases did not contain information on some determinants of breast cancer including reproductive history, family history of breast cancer, anthropometric variables and lifestyle factors. However, residual confounding is unlikely to be a concern in our study since previous studies of the SSRI-breast cancer association that have collected information on these factors have demonstrated that they are not a source of confounding bias [19,21,23,33]. In addition, we did not have information on prescription and over-the-counter use of non-steroidal anti-inflammatory drugs (NSAIDs), some of which have been associated with a reduction in breast cancer risk. If NSAIDs were used to a greater extent by SSRI users, then our risk estimates may have been affected by uncontrolled confounding. However, we have no reason to believe that this was the case.

Given that SSRIs are mainly prescribed for the treatment of depression [34], and that there is a hypothesis that the risk of breast cancer may be increased with chronic depression [35,36], it may be contended that residual confounding by depression may have biased the results towards finding an association between long-term SSRI use and breast cancer. However, since our results demonstrate no increased risk, this source of confounding "by indication" is of less concern. Further, physician prescribing practices for SSRIs are unlikely to be biased by the presence of established risk factors for breast cancer, such as family history and reproductive factors, since an association between the use of SSRIs and breast cancer has not been firmly established.

## Conclusions

In conclusion, few epidemiologic studies have investigated the long-term safety of SSRIs or considered a latent effect of SSRI use on breast cancer risk. Using precise and objective SSRI exposure measures of long-term use that took into account the duration, dosage and timing of SSRI use, our results do not indicate that the risk of breast cancer is increased with short- or long-term use of SSRIs or provide evidence of a latent effect of past SSRI use. Given the potential health benefits of using SSRIs, our results have important implications within the realm of clinical medicine, suggesting that the issue of breast cancer risk may no longer be a concern for women requiring long-term use of SSRIs.

## Abbreviations

CI: Confidence Interval; DDD: Defined Daily Doses; HSN: Health Services Number; HT: Hormone Therapy; NSAIDS: Non-steroidal Anti-inflammatory Drugs; OCS: Oral Contraceptives; OR: Odds Ratio; PRS: Population Registry System; SCA: Saskatchewan Cancer Agency; SD: Standard Deviation; SK: Saskatchewan; SPDP: Saskatchewan Prescription Drug Plan; SSRIS: Selective Serotonin Reuptake Inhibitors.

## Competing interests

All authors (Drs. Janet Ashbury, Linda Lévesque, Kristan Aronson and Ms. Patty Beck) have no conflicts of interest, or financial or other relationships to declare that may influence or bias this work.

## Authors' contributions

Each author contributed to this manuscript. JA analyzed the data and wrote the manuscript. LL contributed substantially to the statistical analysis and interpretation of the data, and to the manuscript organization and editing. PB coordinated all aspects of data collection involving Saskatchewan personnel including data retrieval, linkage procedures and quality control. KA contributed to the conception and design of the study and the on-going progress of the study. All authors reviewed and revised the manuscript.

## Pre-publication history

The pre-publication history for this paper can be accessed here:

http://www.biomedcentral.com/1741-7015/8/90/prepub
